# Influence of Se/N Codoping on the Structural, Optical, Electronic and Photocatalytic Properties of TiO_2_

**DOI:** 10.3390/molecules22030414

**Published:** 2017-03-07

**Authors:** Yelda Y. Gurkan, Esra Kasapbasi, Nazli Turkten, Zekiye Cinar

**Affiliations:** 1 Department of Chemistry, Namik Kemal University, 59030 Tekirdag, Turkey; yyalcin@nku.edu.tr; 2 Department of Molecular Biology and Genetics, Halic University, 34220 Istanbul, Turkey; esrakasapbasi@halic.edu.tr; 3 Department of Chemistry, Yildiz Technical University, 34220 Istanbul, Turkey; cinarz@yildiz.edu.tr

**Keywords:** TiO_2_, DFT calculations, Se/N-codoping, sunlight, heterogeneous photocatalysis

## Abstract

Se^4+^ and N^3−^ ions were used as codopants to enhance the photocatalytic activity of TiO_2_ under sunlight irradiation. The Se/N codoped photocatalysts were prepared through a simple wet-impregnation method followed by heat treatment using SeCl_4_ and urea as the dopant sources. The prepared photocatalysts were well characterized by X-ray diffraction (XRD), X-ray photoelectron spectroscopy (XPS), UV-diffuse reflectance spectroscopy (UV-DRS), scanning electron microscopy (SEM) and Raman spectroscopy. The codoped samples showed photoabsorption in the visible light range from 430 nm extending up to 580 nm. The photocatalytic activity of the Se/N codoped photocatalysts was evaluated by degradation of 4-nitrophenol (4-NP). The degradation of 4-NP was highly increased for the Se/N codoped samples compared to the undoped and single doped samples under both UV-A and sunlight irradiation. Aiming to determine the electronic structure and dopant locations, quantum chemical modeling of the undoped and Se/N codoped anatase clusters was performed using Density Functional Theory (DFT) calculations with the hybrid functional (B3LYP) and double-zeta (LanL2DZ) basis set. The results revealed that Se/N codoping of TiO_2_ reduces the band gap due to mixing of N2p with O2p orbitals in the valence band and also introduces additional electronic states originating from Se3p orbitals in the band gap.

## 1. Introduction

In the last few decades, TiO_2_ has gained an enormous interest due to its potential application in photocatalysis, solar cells and waste remediation. TiO_2_-mediated photocatalysis is an efficient and economic method to eliminate recalcitrant contaminants from water or air, because it is non-energy intensive, operates at ambient conditions and able to mineralize organic pollutants using only atmospheric oxygen as the additional chemical species [1,2,3,4]. Owing to its high chemical and photo stability, environmental friendliness, water insolubility, low-cost, non-toxicity and high oxidative power, TiO_2_ has been proven to be the most efficient photocatalyst for this process [5,6,7,8].

The photocatalytic reactions on TiO_2_ are initiated by band-gap excitation and subsequent generation of electron/hole (e^−^/h^+^) pairs that can initiate redox reactions on the surface. Electrons are trapped at surface defect sites (Ti^3+^) and removed by reactions with adsorbed molecular O_2_ to produce superoxide anion radical O_2_^•−^, while holes react with adsorbed water molecules or OH^−^ ions to produce ^•^OH radicals. ^•^OH radicals are considered to be the principal reactive species responsible for the degradation reactions. However, the wide band-gap of TiO_2_ (~3.2 eV) requires an excitation wavelength that falls in the UV region. This disadvantage of TiO_2_ limits the utilization of solar energy as a sustainable energy source for its excitation because only 5% of the incoming solar energy on the earth’s surface is in the UV-range. In order to utilize natural solar light in TiO_2_ photocatalysis, the band gap of TiO_2_ must be reduced to be active under visible light irradiation. Recombination of photogenerated charge carriers is another major drawback associated with TiO_2_. The majority of the e^−^/h^+^ pairs generated upon band gap excitation are lost through recombination instead of being involved in redox processes at the surface. The e^−^/h^+^ recombination process not only decreases the quantum yield but also decreases the oxidation capability of TiO_2_ [8,9]. Therefore, in recent years research on TiO_2_ has been focused on extending its optical absorption to the visible region of the spectrum in order to substitute UV-light by sunlight and also to increase its photocatalytic activity by decreasing the recombination rate of the charge carriers.

In the past decades, considerable efforts have been devoted to modify the electronic structure of TiO_2_. The most common method is doping in which impurities are introduced into the TiO_2_ matrix in order to reduce the band gap. The dopants develop electronic energy levels within the band gap for absorption of photons or contribute electrons to the valence band (VB). The dopants also behave as trapping sites for electrons and holes to significantly reduce the recombination processes thus prolonging the lifetime of the charge carriers. Metal ion doped TiO_2_ photocatalysts have been extensively studied and found to enhance photocatalytic activity in the visible range [10]. However, some investigators have reported that doping with metal ions enhances the photocatalytic activity while some research groups have found that the presence of cations in TiO_2_ is detrimental for the photocatalytic degradation reactions of organics in aqueous systems [11,12,13,14,15,16]. Moreover, thermal instability and increase in the charge carrier recombination centers have caused metal ion dopants to be unfavorable. Therefore, non-metal doping of TiO_2_ has gained considerable attention as an approach to overcome the drawbacks of metal doping.

Non-metal C, N, S, F, B-doped TiO_2_ photocatalysts have been found to show a relatively high level of activity under visible-light irradiation [17,18,19,20,21,22]. These anion dopants either reduce the band gap of TiO_2_ through mixing their p orbitals with O2p orbitals or introduce additional energy levels into the band gap. Nitrogen seems to be more attractive than all the other anionic dopants because of its comparable atomic size with oxygen, small ionization energy and stability. After Asahi et al. [23] have reported that nitrogen doping of TiO_2_ extends its light absorption to visible light range, nitrogen-doped (N-doped) TiO_2_ has been extensively studied [24,25,26,27,28,29]. However, at high dopant concentrations, the impurity levels in the non-metal doped TiO_2_ act as charge recombination centers and reduce photoactivity.

Recently, it has been reported that the photocatalytic activity of TiO_2_ doped with non-metals can be further increased by the presence of a non-metal ion as a codopant [30]. Codoping of TiO_2_ has exhibited significant improvement in photocatalytic activity as compared to single doping due to synergistic effects of two different non-metals. Yu et al. [31] have investigated N/S-codoped TiO_2_ and obtained a high day-light induced photocatalytic activity. Wang et al. [32] have synthesized N/C-codoped TiO_2_ by a solvothermal method and reported that the surface of TiO_2_ was modified by both C and N via formation of Ti-C bonds, carbonate species and oxynitrides. They have evaluated the photocatalytic activity of their samples by investigating the degradation reaction of bisphenol. Li et al. [33] have obtained high visible light activity for N/F-codoped TiO_2_. In another study, visible light activated TiO_2_ with N and F codopants have been prepared by the surfactant assisted sol-gel method and immobilized on glass substrates [34]. The prepared films have been examined for the oxidation of NO and the modified catalysts have exhibited significant photocatalytic activity under daylight illumination. B and N codoped titania photocatalyst has been synthesized by Ling et al. [35]. The results of their study have shown that the codoping of B and N played an important role in the band gap decrease, which led to the rise of the photocatalytic activity.

Although codoping with two non-metals has been believed to be superior to single doping, codoping at two anionic sites induces significant crystal distortion and charge unbalance resulting in a high recombination rate of the charge carriers [36]. Therefore, more recently, most researchers have concentrated on codoping with metal and non-metal combinations. In this case, the former contributes to the VB while the later forms additional levels in the band gap. N/V-codoped TiO_2_ has been investigated and found that codoping with V and N induces isolated energy levels near the conduction band (CB) and VB causing an effective narrowing of the band gap [37]. Kubacka et al. [38] have synthesized micro-crystalline W/N-codoped TiO_2_ that showed high activity under sunlight. The structural and electronic properties of the codoped photocatalyst have been explored by combining spectroscopic data with Density Functional Theory (DFT) calculations. Effect of metal ions (Fe, Ni, Ag, Pt) on the physicochemical properties of N-doped TiO_2_ has been investigated experimentally [36]. A negative effect of Fe and Ni was observed while Ag and Pt codopants have positive effects.

In our previous study [39], we doped TiO_2_ with Se^4+^ ions. Characterization techniques showed that Se^4+^ is in O–Se–O linkages in the crystal lattice. The absorption threshold of the Se^4+^-doped photocatalyst shifted to the visible region of the spectrum. We obtained a higher photocatalytic activity for the degradation of 4-nitrophenol (4-NP) for the Se^4+^-doped TiO_2_ compared to the undoped TiO_2_. However, we did not observe any direct correlation between the visible light activity and the photocatalytic activity of the doped samples. Our DFT calculations indicated that Se^4+^-doping of TiO_2_ does not cause a significant change in the positions of the band edges; in contrast, it produces additional electronic states originating from the Se 3p orbitals in the band-gap. The visible-light photocatalytic activity of the Se^4+^-doped TiO_2_ is due to these localized mid-gap levels. In one of our earlier studies [40], we doped TiO_2_ with N^3−^ ions. Characterization techniques showed that nitrogen anions are in O–Ti–N linkages and the dopant nitrogen led to an important reduction in the band-gap through substitutional N-doping. We obtained a higher photocatalytic activity for the degradation of 4-NP. Our DFT calculations indicated that band gap reduction arises from the contribution of N 2p to the O 2p and Ti 3d states in the VB of TiO_2_.

Based on these results, we attempted to dope TiO_2_ with Se^4+^ and N^3−^ ions simultaneously to obtain a more active, visible-light driven photocatalyst. This paper has the purpose of determining the electronic structure, optical and photocatalytic properties of Se/N-codoped TiO_2_, to elucidate the chemical nature, the position and the synergistic effect of the dopants on the activity of the photocatalyst. For this purpose, a combination of experimental and quantum mechanical methods were used. In the experimental part of the study, a series of Se/N-codoped TiO_2_ photocatalysts were prepared by means of a simple wet impregnation method and characterized by structural techniques. The photocatalytic activity of the Se/N-codoped TiO_2_ was also determined by investigating the kinetics of the photocatalytic degradation of 4-NP in the presence of the undoped and Se/N-codoped TiO_2_. Modeling of the undoped and Se/N-codoped clusters was performed using DFT calculations to provide a framework for the interpretation of the experimental data and to elucidate the structural and electronic properties of the Se/N-codoped titania.

## 2. Experimental and Computational Details

### 2.1. Materials

TiO_2_ Evonik P-25 grade (Degussa Limited Company, Istanbul, Turkey) with a particle size of about 21 nm and a surface area of 50 m^2^·g^−1^ was used as the photocatalyst without further treatment. Evonik P-25 powder, which is a mixture of anatase and rutile phases (80% anatase, 20% rutile) was chosen as the precursor for Se/N-codoping, in order to compare the results with the previous ones. Moreover, Evonik P-25 is the standard photocatalyst with high activity and has a well-known structure and photocatalytic data. SeCl_4_, urea and 4-NP were purchased from Merck (Istanbul, Turkey). All the chemicals that were used in the experiments were of laboratory reagent grade and used as received without further purification. The solutions were prepared with doubly distilled water.

### 2.2. Preparation of Se/N-Codoped TiO_2_

Doping was performed by an incipient wet impregnation method in order to prevent penetration of the dopant ions into the bulk of TiO_2_, since bulk doping increases the recombination rate of charge carriers resulting in a decrease in photocatalytic activity. SeCl_4_ was used as the Se-source and urea as the N-source. 10 g TiO_2_ Evonik P-25 was mixed with 10 mL of aqueous solutions of SeCl_4_ and urea and stirred at room temperature for 1 h. During this period, the mixture changed color into a pinkish-beige depending upon the dopant concentration. Five different Se/N-codoped photocatalysts containing (wt. %) 0.1 N–0.25 Se, 0.25 N–0.1 Se, 0.5 N–0.5 Se, 0.25 N–0.25 Se, 0.1 N–0.1 Se were prepared. Then, the prepared photocatalysts were washed with water and centrifugally separated three times, heat-treated at 378 K for 24 h to eliminate water, calcined at 623 K for 3 h, ground and sieved. Three different temperatures (623, 723 and 823 K) and three different times (1, 3 and 5 h) were applied to the sample containing 0.5% N–0.5% Se in order to determine the effects of calcination temperature and period on the structure of the photocatalyst.

### 2.3. Characterization Techniques

In order to determine the effect of Se/N-codoping on the crystal structure of TiO_2_, X-ray Diffraction (XRD) patterns were obtained. XRD measurements were carried out at room temperature by using a Philips Panalytical X’Pert Pro X-ray (Philips, Eindhoven, The Netherlands) powder diffraction spectroscope with Cu Kα radiation (λ = 1.5418 A). The accelerating voltage and emission current were 45 kV and 40 mA respectively. The scan ranged from 20 to 70 (2 theta degree) with a scan rate of 3°·min^−1^. Crystallite size was determined using the Scherrer equation:

(1) $$d=\frac{\left(0.9\lambda 180\right)}{\left(\pi FWHM_{hkl}\cos\theta \right)}$$

where *FWHM_hkl_* is the full width at half-maximum of an *hkl* peak at *θ* value. The crystal structure was further analyzed by Raman spectroscopy. Raman spectra were acquired by a PerkinElmer 400F dispersive Raman spectrometer (Perkin Elmer, Waltham, MA, USA) equipped with dielectric edge filters and a cooled CCD detector. Samples were excited using a near infrared 765 nm laser pulse. To examine the morphological structure of the Se/N-codoped TiO_2_ photocatalysts, scanning electron microscopy (SEM) was performed on gold-coated samples by using a SEM apparatus (JEOL JSM 5410 LV, Peabody, MA, USA) operated at an accelerating voltage of 10 kV. The UV-visible diffuse reflectance spectra (UV-DRS) were recorded on a Perkin Elmer Lambda 35 spectrometer equipped with an integrating sphere assembly using BaSO_4_ as the reference material. The analysis range was from 200 to 800 nm. Surface properties of the codoped samples were examined by X-ray photoelectron spectroscopy (XPS). XPS measurements were performed on a SPECS ESCA (Berlin, Germany) system with MgKα source (hν = 1253.6 eV) at 10.0 kV and 20.0 mA respectively. All the binding energies were referenced to the C 1s peak at 284.5 eV. Gaussian/Lorentzian peak shapes were utilized for curve fitting.

### 2.4. Photocatalytic Experiments

The performance of the Se/N-codoped TiO_2_ was assessed on 4-NP by carrying out the photocatalytic degradation reactions under both UV-A and sunlight irradiation. The photocatalytic activity experiments were carried out in a Pyrex double-jacket photoreactor. A water bath connected to a pump was used to maintain the reaction temperature constant. 5 × 8 W blacklight fluorescent lamps emitting light between 300 and 400 nm with a maximum at 365 nm were used as the light source for UV-A irradiation. Total photonic fluence was determined by potassium ferrioxalate actinometer [41] as 3.1 × 10^−7^ Einstein·s^−1^. The experiments under solar light were performed in the second week of May (the outside temperature was 29 °C) in Istanbul (41°02’ latitude, 28°97’ longitude). The daily average solar light intensity was 650 W/m^2^.

In the experiments, a stock solution of 4-NP at a concentration of 1.0 × 10^−2^ mol·L^−1^ was used. The suspension was prepared by mixing specific volumes of this solution containing the desired amount of 4-NP with TiO_2_ Evonik P-25 and the Se/N-codoped TiO_2_. The suspension was agitated in an ultrasonic bath for 15 min in the dark before introducing it into the photoreactor, to ensure adsorption equilibrium between the photocatalyst and 4-NP. The concentration of 4-NP was constant before irradiation. The volume of the suspension was 600 mL. The amount of the photocatalyst used was 0.2 g/100 mL, which was determined as the corresponding optimum photocatalyst concentration. The suspension was stirred mechanically throughout the reaction period in order to prevent TiO_2_ particles from settling. The temperature of the reaction solution was 23 ± 2 °C. Under these conditions, the initial pH was at the natural pH of 4-NP, 5.8 ± 0.1 as measured by a pH-meter (Metrohm 632, Istanbul, Turkey). Duplicate experiments were performed unless otherwise stated.

All the samples, each 10 mL in volume were taken intermittently for analysis. The samples were then filtered through 0.45 μm cellulose acetate filters (Millipore HA, Istanbul, Turkey). The concentration of 4-NP was measured by a UV-Visible spectrophotometer (Agilent 8453, Santa Clara, CA, USA) at 318 nm which was the wavelength of maximum absorption of 4-NP. The calibration curves were prepared for a concentration range of (1.0–10.0) × 10^−5^ mol·L^−1^ and the detection limit for 4-NP was calculated to be 3.79 × 10^−6^ mol·L^−1^. In the experiments, the pH of the reaction solution decreased slightly. For 120 min of degradation the change in the pH was ±0.1, which did not affect the wavelength of maximum absorption in the UV-spectrum of 4-NP.

### 2.5. Computational Models and Methodology

Quantum mechanical modeling techniques were employed in order to determine the effect of the codopants Se^4+^ and N^3−^ on the electronic and optical properties of TiO_2_. Of the two theoretical modeling techniques used for crystalline solids and surfaces, localized modeling technique was used in this study, since dopant ions in crystals are localized. This technique describes small representative portions of the crystal by molecular orbitals.

The anatase phase is the most abundant phase of Evonik P-25 powder. (001) surface is known to have the highest stability and photocatalytic activity among the low index planes of anatase [42]. Therefore, in order to determine the location and the bonding status of the dopant ions, the non-defective anatase (001) surface was modeled with saturated, finite, neutral, and stoichiometric cluster models, cut from the anatase bulk structure. For the free cluster models “water saturation technique” was used in order to avoid spin localization and boundary effects [43].

Two different sized cluster models were considered. The primitive cluster Ti_7_O_18_H_8_ was constructed by using the structure of the anatase unit cell [44]. The primitive cell was then enlarged by extending the lattice vectors resulting in a supercell Ti_25_O_55_H_10_ with 4 × 2 × 1 repetitive units respectively. The construction and the properties of the two undoped TiO_2_ clusters have been reported and explained in detail previously [39].

In the Se/N codoped models, substitutional locations of Se^4+^ ion were analyzed. The structures of the codoped models were constructed by replacing one titanium atom by one selenium atom. For the codopant N, both substitutional and interstitial locations were analyzed. For substitutional models one oxygen was replaced by one nitrogen. In the interstitial model, one nitrogen was added and one OH group was removed. In order to keep the number of atoms the same as in the substitutional model, an oxygen vacancy was also created by using a dummy atom. The anatase surface is Lewis acidic due to the presence of adsorbed water molecules. Water adsorption on anatase surface occurs mostly by dissociative adsorption. Therefore, in the clusters developed, the unsaturated oxygen atoms were terminated with hydrogens and titanium atoms with OH groups, in order to saturate the free valence at the surface and also to keep the average coordination of the surface cluster atoms the same as that in the bulk.

All the calculations were carried out using the Density Functional Theory DFT method within the GAUSSIAN 09 package [45]. The DFT calculations were performed by the hybrid B3LYP functional which combines Hartree-Fock (HF) and Becke exchange terms with the Lee-Yang-Parr correlation functional. The double-zeta LanL2DZ basis set was used in order to take the relativistic effects into account. The dopant positions were optimized by changing their locations in the clusters to find the lowest energy configuration. Optimized geometries of the clusters were calculated to obtain the geometric parameters, the band edges and the band gap energies E_g_ of the undoped and Se/N-codoped photocatalysts.

## 3. Results and Discussion

### 3.1. Crystal Structure

**Figure 1:**
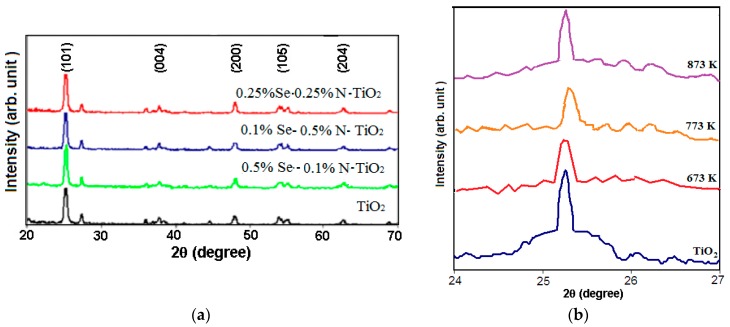
(**a**) X-ray Diffraction (XRD) diffractograms for undoped and 0.5% Se–0.5% N-codoped TiO_2_; (**b**) XRD peaks for (101) planes of undoped and 0.5% Se–0.5% N-codoped TiO_2_.

Figure 1a shows XRD diffractograms of the undoped and Se/N-codoped TiO_2_ samples containing 0.5% Se–0.5% N. The XRD diffractogram of the undoped TiO_2_ (Evonik P-25) shows the presence of both anatase and rutile phases. XRD diffractograms of the Se/N-codoped TiO_2_ have typical peaks of anatase and rutile without any detectable dopant-related peaks. This result reveals that neither Se^4+^ ions nor N^3−^ react with TiO_2_ to form new crystalline phases, the dopants may have moved into the substitutional or interstitial sites of the TiO_2_ crystal structure. The peaks for Se/N-codoped TiO_2_ samples show peak broadening with the dopant-content, which indicates a reduction in the crystallite size and a higher disorder or defectiveness of the crystallites, since doping can lead to formation of new defects and disorder in the particles. The average crystallite sizes of the samples were estimated using the Scherrer equation and presented in Table 1.

A slight shift in the peak position corresponding to (101) plane of anatase to a higher angle was observed as displayed in Figure 1b. This finding indicates that the crystal is distorted by the incorporation of the dopants. Due to a smaller ionic radius (64.0 pm) of Se^4+^ ion than Ti^4+^ ion (74.5 pm) and a higher ionic radius (14.6 pm) of N^3−^ ion than O^2−^ ion (14.0 pm), substitution of Se for Ti and N for O in TiO_2_ crystal lattice resulted in a decrease in the interplanar distance. In addition, a smaller shift in the peak position corresponding to (004) plane of anatase was observed. This shift suggests a slight lattice variation in the vertical direction also. It can also be seen from Table 1 that crystallite size increases with the calcination temperature. The reason may be attributed to the fact that calcination at high temperatures or in long periods causes the doped ions to be desorbed.

**Figure 2:**
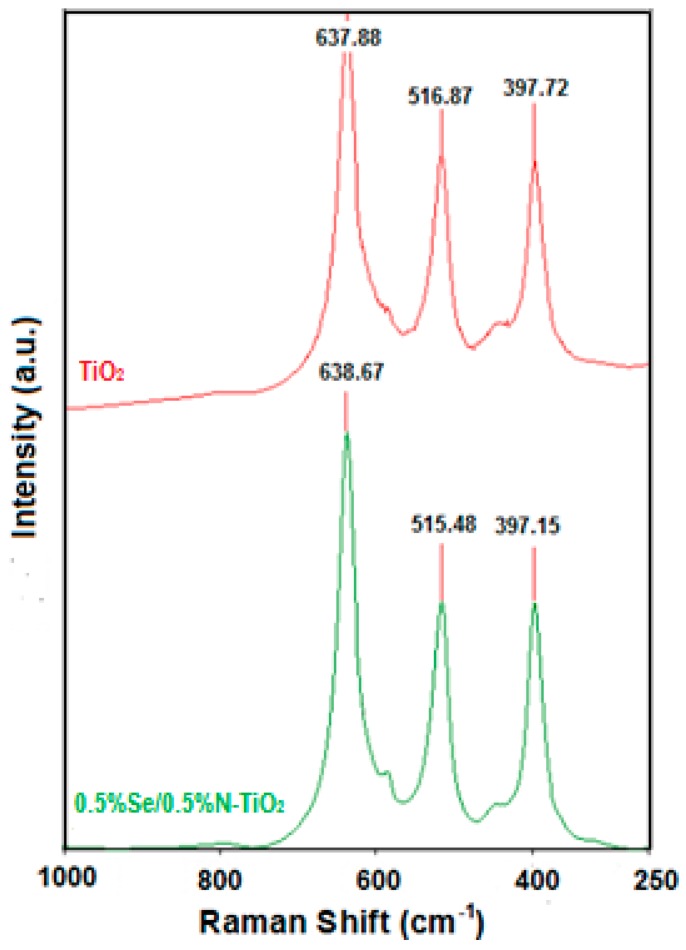
Raman spectra for the undoped and 0.5% Se–0.5% N-codoped TiO_2_.

Raman spectra of the undoped and Se/N codoped samples in Figure 2 support XRD results. Three well-resolved Raman peaks at 398 (B_1g_), 516 (E_g_) and 638 (E_g_) cm^−1^ in the spectra of all the samples were obtained indicating that anatase nanoparticles are the predominant species. The weak peaks at 447, 612 and 826 cm^−1^ could be assigned to E_g_, A_1g_ and B_2g_ modes in rutile phase respectively. No Raman lines due to other crystalline phases can be observed in the Se/N-codoped sample. Three anatase peaks shifted to lower values, confirming the presence of the dopant ions in the crystal lattice. Generally shifting in Raman spectra is caused by defect structures within the material or changes in grain size. For TiO_2_, defect structures, mostly oxygen vacancies not grain size strongly affect the Raman spectrum by producing shifting [46]. Therefore, it may be concluded that Se/N-codoping increases oxygen vacancies in TiO_2_ lattice.

### 3.2. Morphological Structure

**Figure 3:**
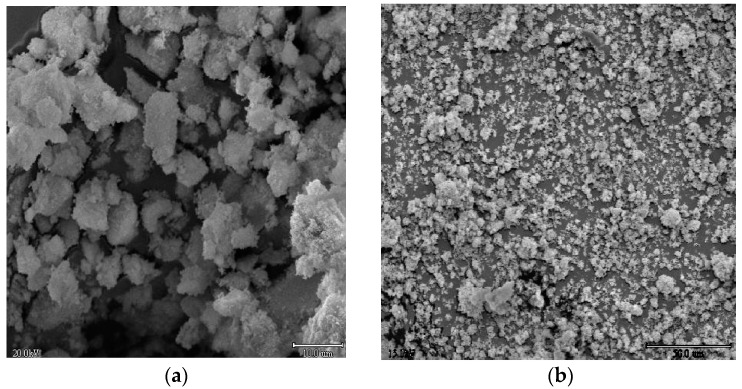
Scanning electron (SEM) micrographs for (**a**) 0.5% Se–0.5% N-codoped TiO_2_; (**b**) undoped TiO_2_.

Figure 3a shows the SEM micrograph obtained for the Se/N-codoped TiO_2_ (0.5% Se–0.5% N). As it can be seen, the sample consists of small, nearly spherical and some larger, elongated particles. SEM micrograph in Figure 3b shows that the undoped TiO_2_ consists of uniform sized spherical particles of around 20–25 μm in diameter. In contrast, the Se/N-codoped TiO_2_ consists of significantly larger particles with an average size of approximately 30–40 μm due to the fact that doping of TiO_2_ causes agglomeration of the crystallites. The tendency of agglomeration may be attributed to the fact that impurity doping leads to the formation of new defects and dislocations in the crystal lattice. The sizes of these aggregates enlarge up to 50 μm.

### 3.3. Optical Absorption and Band Gap Energies

**Figure 4:**
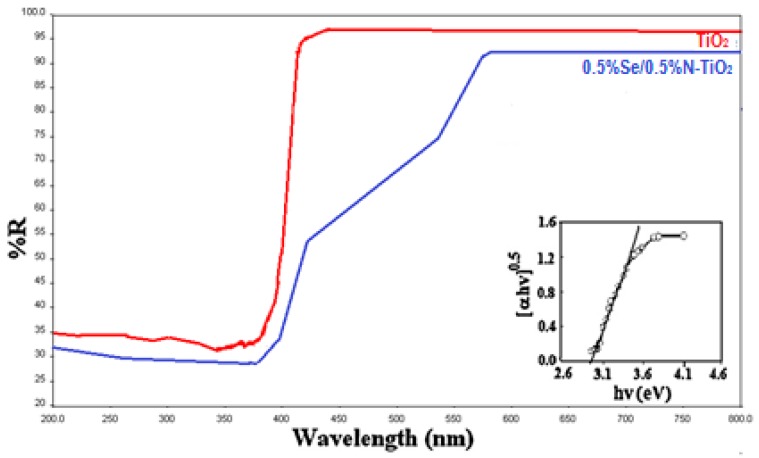
UV-diffuse reflectance (UV-DRS) spectra of the undoped and 0.5%Se-0.5% N-codoped TiO_2_ samples (Red, TiO_2_; blue, Se/N-codoped TiO_2_).

UV-visible diffuse reflectance spectra for the undoped and Se/N-codoped TiO_2_ are displayed in Figure 4. The spectrum for the undoped TiO_2_ has a sharp absorption edge at around 380 nm, however the absorption threshold of the Se/N codoped TiO_2_ shifted towards the visible region of the spectrum. In contrast to the undoped TiO_2_, a high visible light absorption band from ca. 430 nm extending up to ca. 580 nm was obtained, which is consistent with the color of the samples.

In the UV-DRS spectrum of the Se/N-codoped TiO_2_, two optical absorption thresholds were observed, one in the UV-region at around 430 nm, the other in the visible region at 550 nm. The first one is a rather sharp absorption edge indicating that the dopant ions are localized in the TiO_2_ lattice, occupying Ti^4+^ and O^2−^ positions. It can be seen that the codoped sample presents a significant absorption in the visible region between 430–550 nm. In between 550–580 nm, there is a tailing which may be attributed to the presence of mid-gap levels in the band-gap of the codoped TiO_2_.

The band gap energies of the codoped photocatalyst samples were calculated through the use of the Kubelka-Munk formula:

(2) $$F(R)=\frac{{\left(1-R\right)}^{2}}{2R}$$

where *R* is the reflectance read from the spectrum. Using the Tauc equation by plotting [*F*(*R*).hν]^n^ vs. hν, where hν is the photon energy and *n* = 1/2 [47], the band gap energies were deduced from the intersection of the Tauc’s linear portion extrapolation with the photon energy axis as depicted in the insert in Figure 4. The calculated band gap energies and the corresponding wavelengths are presented in Table 1. The values indicate that the absorbance in the visible region of the Se/N-codoped samples increases with the concentration of the dopants in TiO_2_. The presence of both ions caused an even more decrease in the band gap and an increase in the absorption in the visible region as compared to single Se-doped and N-doped TiO_2_ [39,40].

### 3.4. XPS Analyses

**Figure 5:**
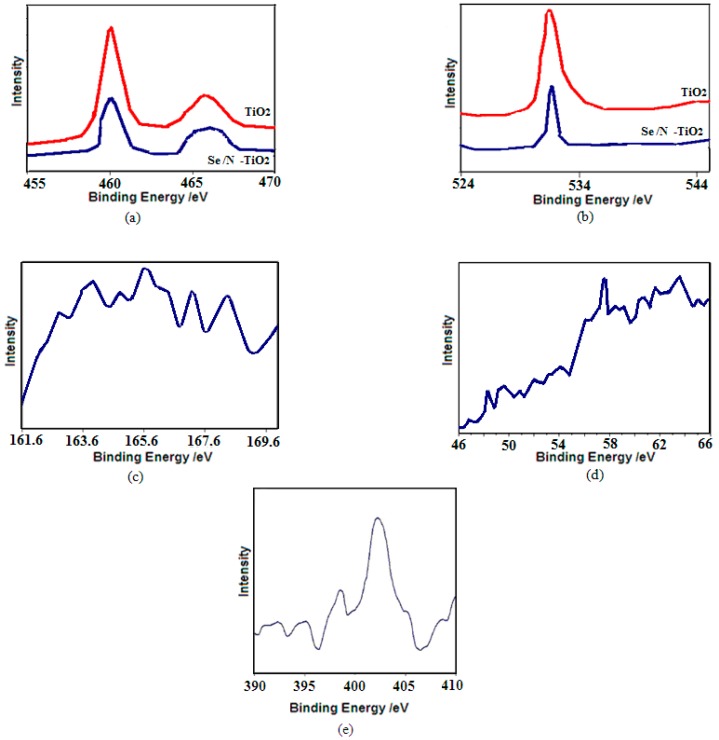
X-ray photoelectron (XPS) spectra of the undoped and 0.5% Se–0.5% N-codoped TiO_2_ samples, (**a**) Ti 2p; (**b**) O 1s; (**c**) Se 3p; (**d**) Se 3d; (**e**) N 1s.

X-ray photoelectron spectroscopy (XPS) was used to examine the bonding and status of the dopants in the Se/N-codoped TiO_2_. Five areas of the XPS spectra, displayed in Figure 5 were examined, Ti 2p region near 460 eV, O 1s region near 530 eV, Se 3p region near 165 eV, Se 3d region near 55 eV and N 1s near 400 eV. In Figure 5a, the two peaks at ca. 460 and 465 eV correspond to the photo-splitting electrons Ti^4+^ 2p_3/2_ and Ti^4+^ 2p_1/2_ indicating that titanium in the sample is in the form of Ti^4+^. In the XPS spectrum of the Se/N-codoped sample, Ti 2p_3/2_ peak appears at 461.1 eV higher than 459.9 eV for the undoped TiO_2_ but lower than 461.3 eV for the Se-doped TiO_2_. The higher binding energy confirms the presence of substitutional Se^4+^ cations in the crystal. Since the electronegativity of Se^4+^ is more than titanium, the electron density around titanium cations decreases causing an increase in the binding energy. On the other hand, the lower binding energy than that for Se-doped TiO_2_ indicates the presence of substitutional and/or interstitial N anions in the same crystal. Since the tendency of nitrogen to attract the bonding electrons toward itself is lower than that of oxygen, the electron density around Ti atoms increases leading to a decrease in the binding energy. The broadness of Ti peaks for the codoped sample may be attributed to the presence of titanium atoms bonded to two different atoms, oxygen and nitrogen.

The O 1s binding energy of the codoped sample is located at 530.8 eV which is assigned to the metallic oxide (O^2−^) in the TiO_2_ lattice. There is a second shoulder peak at 529.9 which corresponds to surface hydroxyl groups. This implies that the oxygen environment is the same as in the undoped TiO_2_ indicating the presence of substitutional Se and N atoms (Ti–O–Se, Ti–N–Ti) rather than interstitial ones (Ti–O–N) in the crystal lattice. The signals of the Se dopant were found to be weaker than Ti and O peaks, due to the low doping level. The peak at 165.6 eV corresponds to Se 3p_3/2_ electrons indicating that Se in the codoped sample is in the form of Se^4+^ [48]. The presence of the peak at 56.1 eV corresponding to Se 3d_5/2_ of Se^4+^ cation confirms this finding [49]. The characteristic 3d_5/2_ peaks at 55.5 eV [50] and 53.0–54.0 eV [51] corresponding to elemental Se and Se^2−^ were not observed. These observations reveal that selenium in the as-prepared sample is in the form of Se^4+^ that can penetrate into the TiO_2_ lattice and substitute Ti^4+^ cations.

The N 1s spectrum in Figure 5e has two peaks at 397.8 and 402.3 eV. The first peak at 397.8 corresponds to anionic N substitutionally incorporated in TiO_2_ in O–Ti–N linkages. The peak is 0.9 eV higher than the characteristic binding energy of 396.9 eV in TiN [52]. Therefore, it may be attributed to the 1s binding energy of the N atom in the environment O–Ti–N. This shift to a higher energy results from the fact that when N substitutes for O in TiO_2_, O–Ti–N structures form, thus the electron density around N is less than that in TiN (N–Ti–N). On the other hand, the second peak at 402.3 eV may be assigned to oxidized N such as the ones in Ti–O–N species as in interstitial doped TiO_2_ or adsorbed NO, NO_2_ species on the surface, since the binding energy is higher than the typical binding energy of 396.9 eV in TiN indicating that the formal charge on the doped N is more positive than the one in TiN [26]. Even though the presence of interstitial N atoms in the prepared Se/N codoped TiO_2_ cannot be ruled out, this peak is likely to result from the formation of nitrogen-containing species such as NO, NO_2_, NO_2_^−^, NO_2_^2−^ adsorbed on the surface.

### 3.5. Photocatalytic Activity

**Figure 6:**
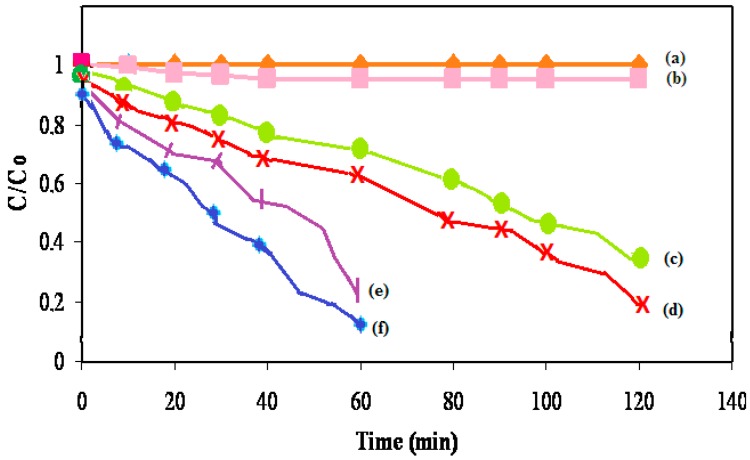
Kinetics of the photocatalytic disappearance of 4-NP on the undoped and Se/N-codoped TiO_2_ (0.5% Se–0.5% N) (**a**) with light; (**b**) with TiO_2_; (**c**) with TiO_2_ + light; (**d**) with Se/N-codoped TiO_2_ + light; (**e**) with TiO_2_ + sunlight; (**f**) with 0.5% Se–0.5% N-codoped TiO_2_ + sunlight.

To explore the photocatalytic activity of the Se/N-codoped TiO_2_ samples, the degradation reaction of 4-NP was investigated in aqueous suspensions under both UV-A and natural solar light irradiation. Figure 6 shows the kinetics of disappearance of 4-NP from an initial concentration of 1.0 × 10^−4^ mol·L^−1^ which was determined as the optimum concentration under four conditions. In non-irradiated suspensions, there was a slight loss, ca. 4.3%, due to adsorption onto TiO_2_ particles. As seen in Figure 6, there was no direct photolysis taking place. The degradation of 4-NP is due entirely to photocatalysis. In the presence of TiO_2_, the concentration change amounts to 70% after irradiating for 120 min. The semi-logarithmic plots of concentration data gave a straight line. This finding indicates that the photocatalytic degradation of 4-NP in aqueous TiO_2_ suspensions can be described by a pseudo-first order kinetic model, ln C = −kt + ln C_0_, where C_0_ is the initial concentration and C is the concentration of 4-NP at time t.

In the presence of Se/N-codoped TiO_2_, the degradation rate of 4-NP increased, as expected. The concentration data gave a straight line, indicating that the kinetics of the degradation reaction of 4-NP in the presence of the Se/N-codoped TiO_2_ also obeys the first-order kinetic model. The Se/N-codoped TiO_2_ also exhibited substantial photocatalytic activity under direct sunlight irradiation, with 90% of 4-NP removed in 60 min as compared to 73% removal with the undoped TiO_2_ and 88% removal with single Se-doped sample. The result is that the prepared codoped samples are photocatalytically active under solar light.

The enhanced photocatalytic activity of the Se/N-codoped TiO_2_ is due to several factors such as; synergistic effect of the dopants, formation of the oxygen vacancies, improved structures and the enhanced photo absorption. Due to their favorable energy levels (2.27 eV), Se^4+^ centers may act either as electron or hole traps so that charge carriers are temporarily separated. On the other hand, substitutional N^−3^ inhibits e^−^/h^+^ recombination due to charge compensation between N^3−^ and Ti^4+^. Thus, the lifetime of the charge carriers increases leading to an enhancement of the photocatalytic activity. The role of the dopant nitrogen is not only to decrease e^−^/h^+^ recombination rate, but it also induces a substantial reduction of the formation energy of oxygen vacancy on TiO_2_ [24]. This implies that N-doping causes oxygen vacancy formation on the surface of the particles in agreement with the Raman spectrum. The formation of the oxygen vacancies on the surface favors the adsorption of water molecules and thus increases the amount of hydroxyl radicals which are responsible of the degradation of 4-NP.

The high photocatalytic activity of the Se/N codoped TiO_2_ is also due to the fact that it has smaller particle size thus higher adsorption area toward the organic pollutant. Moreover, the increase in the light absorbance extending up to visible light range with Se/N-codoping indicates that more electrons and holes are generated and participate in the surface redox reactions causing an increase in the amount of hydroxyl radicals which are responsible of the degradation of the pollutant molecule.

The results presented in Table 2 show the effect of Se and N concentrations of the codoped photocatalysts on the photocatalytic degradation of 4-NP. As it can be seen from the values, the photocatalytic degradation rate of 4-NP first increased and then decreased passing through the maximum degradation for the photocatalyst containing 0.5% Se and 0.5% N. There appears to be an optimal dopant concentration, 0.5%, above which the observed photoreactivity decreases. The reason may be attributed to the fact that at lower concentrations below the optimal value, photoreactivity increases with an increasing dopant concentration because there are available trapping sites. The dopants provide more trap sites for electrons and holes in addition to the surface trap sites, adsorbed O_2_ and OH^−^. However, at high dopant concentrations, the photocatalytic activity of the codoped samples decreased. This is because the recombination rate of the charge carriers increases exponentially with the dopant concentration. The average distance between trap sites decreases with increasing the number of dopants confined within a particle. Thus, it may be concluded that the number of trapped carriers is the highest in 0.5% Se–0.5% N codoped sample for which the highest photoreactivity was obtained.

In addition, the experiments demonstrated that there is no direct correlation between the visible light activity and the photocatalytic activity. The optimum dopant concentration was found to be 0.5% Se–0.5% N. However, the UV-DRS spectrum of this sample revealed intermediate values of the band-gap.

### 3.6. Electronic Structures

**Figure 7:**
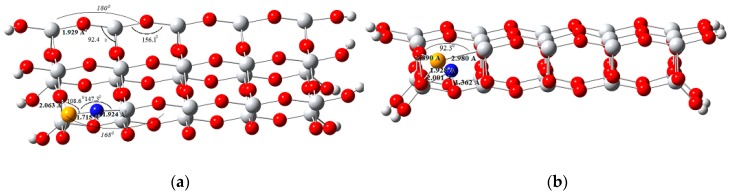
Optimized structures of Se/N-codoped TiO_2_ clusters (**a**) substitutional Se/N-codoped model; (**b**) interstitial Se/N-codoped model (Grey, Ti; red, O; orange, Se; white, H; blue, N).

The structures obtained for the undoped and Se/N-codoped TiO_2_ cluster models are presented in Figure 7. Electronic structure calculations of the models gave structures with deviations, which are not as symmetrical as that of the undoped TiO_2_ model. The results indicate that the size and electronegativity difference between the two codopants induce structural changes.

(001) surface of the undoped TiO_2_ cluster contains the four- and five-fold-coordinated titanium atoms representing Lewis acid sites and the two- and three-fold-coordinated oxygen atoms which act as Lewis base sites. Site preferences of the dopants on (001) surface were determined by calculating the total energies of the codoped clusters. The results indicate that for Se^4+^ four-fold-coordinated Ti site substitution is favored over five-fold-coordinated Ti site substitution by ~36 kcal·mole^−1^. For substitutional nitrogen, two-fold-coordinated O site is favored over three-fold-coordinated O site, and nitrogen prefers to be at the position closest to Se^4+^. In the interstitial model, the optimum position for the vacancy was found to be the one near Se^4+^ dopant.

**Figure 8:**
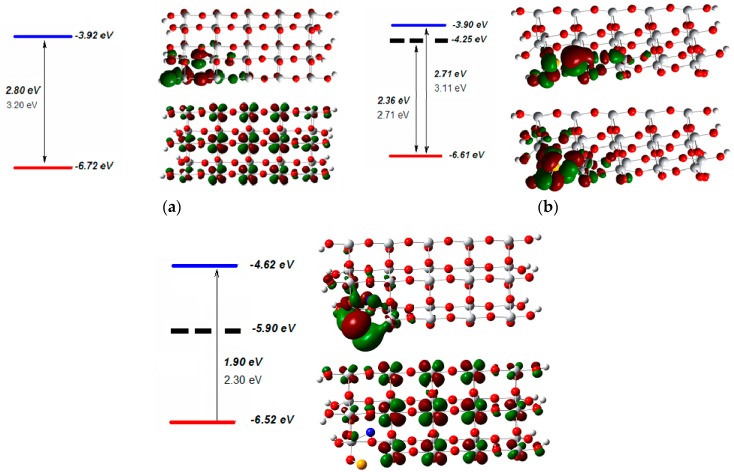
Energy level diagrams and the frontier orbitals of the (**a**) undoped TiO_2_; (**b**) substitutional Se/N-codoped TiO_2_; (**c**) interstitial Se/N-codoped TiO_2_ clusters computed with DFT/B3LYP method. (Grey, Ti; red, O; orange, Se; white, H; blue, N) (Values in italics are the DFT results).

The visible light activity of a photocatalyst depends upon the magnitude of the band-gap and the presence or absence of any intermediate electronic states within the band-gap. On the other hand, the photocatalytic activity of TiO_2_ is governed by the positions of the band edges. A schematic diagram of the electronic energy levels for the undoped and Se/N-codoped anatase models obtained from electronic structure calculations are presented in Figure 8. For the clusters developed in this study, the energies of the highest occupied HOMO and the lowest unoccupied molecular orbitals LUMO were used to represent the VB and CB edges, while the occupied and unoccupied molecular orbitals correspond to the electronic states in the VB and CB respectively. An examination of the calculated band-gap energies of the undoped and codoped clusters in Figure 8 shows that the DFT/B3LYP method underestimates the band-gap energy due to the well-known shortcoming of the exchange-correlation potential used within the framework of DFT. The experimental band-gap energy of the undoped TiO_2_ (3.2 eV) was adopted as the benchmark to correct the calculated values. The calculated band-gap was corrected using a scissors operator that displaces the empty and occupied bands relative to each other by a rigid shift of 0.40 eV to bring the minimum band-gap in line with experiment for the band-gap of anatase.

The computational results show that codoping with Se^4+^ and substitutional nitrogen causes a significant change in the position of the valence band edge. The reason is that N 2p states mix with O 2p states and reduce the band gap. For the substitutional model, the calculations indicated the presence of three empty mid-gap levels in the band-gap as shown in Figure 8. These intermediate electronic states were determined to be mainly originating from the Se3p states hybridized with the O 2p states by examining the calculated coefficients of the orbital wave functions. These energy levels are not populated by electrons. They are not donor states but allowed energy states. Thus, they induce a decrease in the band gap as the dopant concentration increases as obtained by UV-DRS analysis. The increase in the concentration of the dopant Se^4+^ introduces more electronic states into the band gap, thus enhances the density of the electronic states in the gap. The presence of these intermediate levels separates the band-gap of the Se/N-codoped TiO_2_ into two parts; a wider lower gap and a significantly narrower upper gap. These intermediate energy levels offer additional steps for the absorption of low energy photons through the excitation of VB electrons to these intermediate energy levels, from where they can be excited again to the CB. The experimentally observed absorptions in the range 430–550 nm and 550–580 nm and the rather diffused character of the UV-DRS spectrum of the Se/N-codoped TiO_2_ samples may be attributed to the excitation of electrons to or from these additional electronic levels. The lower gap was calculated to be 2.71 eV corresponding to a 458 nm photon which is in agreement with the experimental results obtained from the UV-DRS spectra of the codoped samples. Therefore, it may be stated that the lower gap is responsible for the absorption in the first region of the spectrum between 430–550 nm, while the second region between 550–580 nm corresponds to the excitation of electrons from mid-gap levels to the CB.

On the other hand, in the interstitial model, N 2p states mix with Se 3p orbitals and thus form a mid-gap level between the VB and CB of TiO_2_. The contribution of Se 3p orbitals to the lowest unoccupied orbital was found to be less than the one in substitutional model. Although we may not rule out the presence of interstitial nitrogens, the codopant N is in O–Ti–N structures while Se ion substitutes for Ti in our samples. Moreover, comparison of the energies of the two models indicated that substitutional model is more stable than interstitial model.

## 4. Conclusions

Codoping of TiO_2_ with Se^4+^ and N^3−^ ions was performed through a simple wet-impregnation method using SeCl_4_ and urea as the dopant sources. The characterization results reveal that Se^4+^ is in O–Se–O while N^3−^ is in O–Ti–N linkages in the crystal lattice. The Se/N codoped samples showed photoabsorption in the visible light range from 430 nm extending up to 580 nm. The degradation of 4-NP was highly increased for the Se/N codoped samples compared to the undoped and single doped samples under both UV-A and sunlight irradiation. The enhanced photocatalytic activity of the codoped samples may be attributed to the increase in the number of trap sites for electrons and holes, increase in the photoabsorption, smaller particle size and the formation of oxygen vacancies on the surface. The experiments demonstrated that there is no direct correlation between the visible light activity and the photocatalytic activity. 623 K, 3 h and 0.5% Se–0.5% N were determined to be the most suitable calcination temperature, calcination period and the codopant concentration to prepare the photocatalyst with the highest photocatalytic activity. Eventually, on the basis of experimental results combined with DFT calculations, it may be concluded that Se/N-codoping of TiO_2_ reduces the band gap due to mixing of N 2p with O 2p orbitals in the VB and also introduces additional electronic states originating from the Se 3p orbitals in the band gap.

## Figures and Tables

**Table 1:** Crystallite sizes, band gap energies E_g_ and absorption wavelengths λ for the undoped and Se/N-codoped TiO_2_ samples.

Samples	Calcination Temperature (K) ^1^	Crystallite Size (nm)	λ (nm)	Eg (eV)
TiO_2_ Evonik P-25	623	22.3	411	3.01
0.25% Se–0.1% N	623	19.0	453	2.73
	723	19.3	442	2.80
	823	19.2	437	2.83
0.1% Se–0.25% N	623	17.9	460	2.69
	723	18.5	455	2.72
	823	19.0	451	2.74
0.5% Se–0.5% N	623	16.8	473	2.62
	723	17.4	467	2.65
	823	17.9	458	2.70
0.25% Se–0.25% N	623	17.3	482	2.57
	723	17.6	476	2.60
	823	17.9	469	2.64
0.1% Se–0.1% N	623	19.6	495	2.50
	723	20.1	488	2.54
	823	20.4	480	2.56

^1^ All the values are for a calcination period of 3 h.

**Table 2:** Apparent first order rate constants k for the photocatalytic degradation of 4-NP in the presence of the Se/N-codoped TiO_2_ samples.

Photocatalyst	k (10^−3^·min^−1^)	r	% Degradation
TiO_2_Evonik P-25	9.21 ± 0.009	0.991	69.83
	*14.15 ± 0.008* ^1^	*0.996*	*73.15*
0.25% Se–0.1% N	14.85 ± 0.005	0.991	77.19
	*17.21 ± 0.009*	*0.998*	*79.83*
0.1% Se–0.25% N	17.52 ± 0.008	0.985	79.58
	*18.89 ± 0.002*	*0.982*	*81.93*
0.5% Se–0.5% N	20.21 ± 0.007	0.994	87.71
	*23.37 ± 0.006*	*0.990*	*89.25*
0.25% Se–0.25%N	18.99 ± 0.001	0.987	82.70
	*20.78 ± 0.002*	*0.983*	*85.82*
0.1% Se–0.1% N	14.97 ± 0.003	0.986	73.67
	*16.88 ± 0.001*	*0.995*	*75.17*

^1^ Values in italics are the results of sunlight experiments.
